# Atypical Mycoplasma pneumoniae Infection Presenting With Abdominal Pain in a Four-Year-Old Boy: A Case Report

**DOI:** 10.7759/cureus.84081

**Published:** 2025-05-14

**Authors:** Masazumi Miyahara, Kyoko Osaki, Kazuma Terauchi

**Affiliations:** 1 Department of Pediatrics, Okanami General Hospital, Iga, JPN; 2 Department of Radiology, Okanami General Hospital, Iga, JPN

**Keywords:** abdominal pain, children, extrapulmonary, gastrointestinal, mycoplasma pneumoniae, mycoplasma pneumoniae pneumonia

## Abstract

*Mycoplasma pneumoniae* (*M. pneumoniae*) infections typically affect the respiratory system but can also present with extrapulmonary manifestations, including gastrointestinal involvement. We report the case of a four-year-old boy who presented with abdominal pain and fever, with minimal respiratory symptoms throughout the course of his illness. Despite initial treatment with clarithromycin, his symptoms persisted, and the chest radiographic findings showed worsening pneumonia. Imaging revealed small bowel fluid retention and impaired intestinal motility, suggesting gastrointestinal involvement likely mediated by immune mechanisms. Laboratory findings included elevated lactate dehydrogenase (LDH) levels and mild liver dysfunction, consistent with systemic inflammation. The patient was treated with tosufloxacin and corticosteroids, which led to clinical improvement and symptom resolution. This case highlights the diagnostic challenge posed by atypical presentations of *M. pneumoniae* infection, particularly in young children with minimal respiratory symptoms. Intestinal involvement, although rare, should be considered a potential extrapulmonary manifestation. Elevated LDH levels and poor response to macrolide therapy can indicate refractory or macrolide-resistant cases, necessitating alternative treatments such as corticosteroids and fluoroquinolones. Clinicians should be aware of these atypical presentations to ensure early diagnosis and tailored therapy, improving outcomes in pediatric patients with unusual manifestations of *M. pneumoniae* infections.

## Introduction

*Mycoplasma pneumoniae* (*M. pneumoniae*) is a leading pathogen causing respiratory infections in school-age children and young adults. Although the manifestations of *M. pneumoniae* infection are primarily respiratory, extrapulmonary involvement has been reported in 11-26% of children, affecting systems such as the skin, central nervous system, blood, heart, gastrointestinal tract, and joints [1]. Gastrointestinal symptoms such as vomiting and diarrhea are relatively common, whereas organ-specific complications such as hepatitis, pancreatitis, or intestinal involvement have been far less frequently reported [2,3].

This report describes the case of a four-year-old boy diagnosed with *M. pneumoniae* pneumonia (MPP), presenting predominantly with abdominal pain and minimal respiratory symptoms. Despite the general observation that *M. pneumoniae* infections in children under five years of age tend to manifest as mild upper respiratory tract infections with vomiting and diarrhea [1], this case strongly suggests macrolide-resistant MPP. The abdominal pain was attributed to *M. pneumoniae*-related intestinal involvement with impaired bowel motility. This atypical presentation underscores the diverse extrapulmonary manifestations of *M. pneumoniae* and highlights the diagnostic and management challenges associated with such cases.

## Case presentation

**Figure 1:**
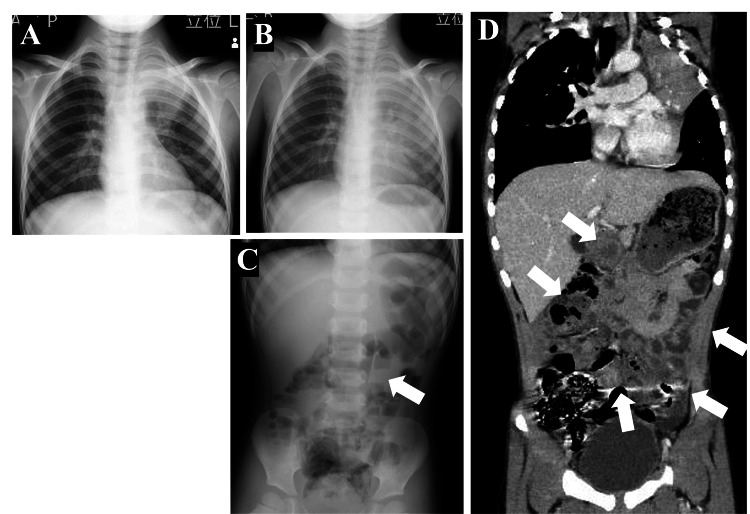
Radiologic findings throughout the clinical course. A: Initial chest radiograph (day five of illness) showing localized consolidation in the left upper lung field, consistent with pneumonia. B: Chest radiograph obtained on admission (day eight) demonstrates progression to diffuse consolidation in the entire left lung, particularly in the upper lobe, with obscuration of the left heart border (silhouette sign). C: Upright abdominal radiograph on admission indicates a step-ladder pattern of air-fluid levels in the small bowel, suggesting the presence of localized ileus (white arrow). D: Contrast-enhanced abdominal CT scan on day 11 shows extensive fluid accumulation in the small bowel (white arrows), reflecting impaired intestinal motility. No evidence of mechanical obstruction was identified.

A four-year-old boy presented with a two-day history of fever (range: 37.7-38.0°C) and intermittent abdominal pain. Abdominal ultrasonography revealed no significant abnormalities, and he was treated symptomatically on an outpatient basis. However, his abdominal pain, of the same intensity, persisted through day five, when he developed a high fever (40°C) accompanied by a mild, non-productive cough. Laboratory tests performed at that time revealed a white blood cell count (WBC) slightly above the upper limit of normal, with mildly elevated C-reactive protein (CRP) and lactate dehydrogenase (LDH) and normal aspartate aminotransferase (AST) and alanine aminotransferase (ALT) levels (Table 1). Chest radiography revealed consolidation in the left upper lung (Figure 1, Panel A). Given the concurrent local outbreak of *M. pneumoniae* infection, a pharyngeal swab was subjected to an *M. pneumoniae* antigen test using the loop-mediated isothermal amplification method; the test yielded a positive result. Clarithromycin therapy was initiated, and the patient continued to be managed as an outpatient. However, his fever and abdominal pain persisted, and oral intake became difficult owing to abdominal discomfort. Consequently, the patient was admitted to the hospital on the eighth day after symptom onset. His medical history was notable for nodular prurigo-type atopic dermatitis. There was no significant family history, including autoimmune, allergic, or respiratory conditions.

**Table 1:** Laboratory findings on day five and eight of illness. WBC: white blood cell count; RBC: red blood cell count; Ch-E: cholinesterase; AST: aspartate aminotransferase; ALT: alanine aminotransferase; LDH: lactate dehydrogenase; CPK: creatine phosphokinase; T-Cho: total cholesterol; BUN: blood urea nitrogen; Crea: creatinine, CRP: C-reactive protein; IgE: immunoglobulin E; LAMP: loop-mediated isothermal amplification

Laboratory parameters	Patient value 1	Patient value 2	Reference range
	Day five of illness	On admission (day eight of illness)	
Peripheral blood test			
WBC	9,300/μL	7,300/μL	4,000–9,000
Neutrophil	71%	67%	39–81
Lymphcyte	21%	21%	16–50
Monocyte	7%	5%	2–10
Eosinophil	1%	7%	2–10
RBC	463 × 10^4^/μL	466 × 10^4^/μL	400–520
Hemoglobin	12.1 g/dL	12.1 g/dL	13.0–17.0
Hematocrit	37.6%	36.6%	38.0–49.0
Platelet	21.9 × 10^4^/μL	26.7 × 10^4^/μL	12.0–44.0
Serum biochemical test			
Total protein	7.2 g/dL	6.5 g/dL	6.5−8.５
Albumin	3.9 g/dL	3.6 g/dL	4.1−5.３
Total bilirubin	0.38 mg/dL	-	0.2−1.2
Ch-E	310 U/L	-	214–466
AST	40 IU/L	124 IU/L	10–35
ALT	10 IU/L	55 IU/L	10–35
LDH	383 U/L	707 U/L	110–225
CPK	81 IU/L	53 IU/L	50–200
T-Cho	-	150 mg/dL	150–219
BUN	10.7 mg/dL	10.0 mg/dL	9.0–22.0
Crea	0.33 mg/dL	0.26 mg/dL	0.50–1.10
Sodium	135 mEq/L	138 mEq/L	138–145
Potassium	4.8 mEq/L	3.9 mEq/L	3.4–4.7
Chloride	98 mEq/L	102 mEq/L	99–108
CRP	0.85 mg/dL	0.52 mg/dL	0.00–0.30
Ferritin	-	482.0 ng/mL	39.9–465.0
IgE	-	2,153 IU/mL	<170
*Mycoplasma pneumoniae* antigen (LAMP)		Positive	Negative

On admission (day eight of illness), the patient was alert but slightly irritable, with a temperature of 37.5°C, heart rate of 152 beats/minute, respiratory rate of 30 breaths/minute, and oxygen saturation of 98% on room air. He appeared fatigued but showed no signs of respiratory distress. On physical examination, no cardiac murmurs were detected, and auscultation of the lungs revealed no abnormal sounds, although air entry was slightly decreased on the left side. The abdomen was flat with no tenderness, hepatomegaly, or splenomegaly. Skin examination showed resolved inflammation from atopic dermatitis that had left behind dark eczematous lesions.

Compared to earlier laboratory findings obtained on day five, on day eight, the WBC decreased to within the normal range, while CRP remained mildly elevated. AST, ALT, and LDH levels showed marked increments. Electrolyte and amylase levels remained within the normal ranges, and IgE levels were significantly elevated (Table 1). Chest radiography obtained on admission demonstrated diffuse consolidation involving nearly the entire left lung, obscuring the cardiac silhouette (Figure 1, Panel B), representing worsening of the initial findings. An upright abdominal plain radiograph taken at the same time revealed partial small-bowel air-fluid levels (Figure 1, Panel C), but no signs of strangulation or extensive paralytic ileus. At that time, the differential diagnoses for the persistent abdominal pain included previously reported extrapulmonary manifestations of *M. pneumoniae* involving abdominal organs as well as nonspecific gastrointestinal symptoms associated with pneumonia and constipation. However, the exact cause of the abdominal pain remained unclear.

Despite concomitant administration of acetaminophen for symptomatic relief, clarithromycin failed to achieve sustained fever resolution after >72 hours, and LDH levels remained elevated. Therefore, clarithromycin was replaced with tosufloxacin, and intravenous prednisolone (1 mg/kg/day) was initiated. During hospitalization, the patient had a fever higher than 38°C on the first night but was afebrile thereafter. Intermittent abdominal pain persisted, and appetite remained poor. On day nine, an enema resolved stool retention. However, abdominal pain continued. Contrast-enhanced chest and abdominal CT performed on day 11 revealed resolution of the pulmonary findings but fluid retention in the small intestine, suggesting impaired intestinal motility (Figure 1, Panel D). The abdominal pain subsided, and the patient’s appetite improved soon after the CT scan, suggesting that the treatment was beginning to take effect at that time. Prednisolone was tapered and switched to oral administration, and eventually discontinued. Tosufloxacin was administered for seven days, and no recurrence of symptoms was observed during outpatient follow-up.

## Discussion

While *M. pneumoniae* infections typically manifest as respiratory symptoms, extrapulmonary manifestations involving various organ systems, which may progress to severe or life-threatening conditions, have been reported. Extrapulmonary manifestations frequently occur without pneumonia, leading to atypical presentations [4]. In our patient, acute abdominal pain with minimal respiratory symptoms presented a diagnostic challenge.

Although instances of *M. pneumoniae* infection causing abdominal pain, such as hepatitis, pancreatitis, splenic infarction, pleural effusion, and retroperitoneal exudation, have been reported [2,3,5,6], these typically present with organ-specific signs or systemic features such as jaundice, abdominal tenderness, or elevated pancreatic enzymes. In contrast, our patient had diffuse, non-localized abdominal pain without laboratory or imaging evidence of organ-specific involvement, and CT imaging revealed only bowel fluid retention suggestive of impaired motility. These findings supported the possibility of immune-mediated intestinal dysfunction, a rarely described extrapulmonary manifestation of *M. pneumoniae* infection. Moreover, some reports indicate that among extra-abdominal diseases causing acute abdomen in children, pneumonia is relatively common [7]. However, such cases typically involve basal pneumonia, where inflammation spreads to the diaphragm, leading to abdominal pain. In our case, the pulmonary lesion was observed in the left upper lung at the onset of abdominal pain, suggesting a different underlying mechanism from previously reported cases. CT findings of small-bowel fluid retention and large-bowel stool retention, combined with the persistence of abdominal pain despite stool evacuation, indicated that constipation alone could not explain the symptoms. The abdominal pain was considered to result from intestinal involvement due to *M. pneumoniae* infection, leading to reduced bowel motility; however, inflammatory findings such as bowel wall thickening were scarce. Clinically, the pain was intermittent and diffuse, without clear localization or peritoneal signs, and not aggravated by movement or palpation. The patient exhibited no signs of guarding or rebound tenderness. Associated vomiting or diarrhea was absent.

The absence of vomiting or diarrhea suggested immune-mediated intestinal involvement rather than direct bacterial invasion. Elevated LDH levels further supported a dysregulated immune response, commonly associated with refractory MPP. The pathogenesis of extrapulmonary *M. pneumoniae* manifestations likely involves the following three mechanisms: (1) direct bacterial effects at the site of inflammation mediated by host cytokine release; (2) indirect effects through autoimmune responses or immune complex formation; and (3) vascular damage mediated by cytokines, chemokines, and immunomodulatory factors [8,9]. In this case, the absence of vascular abnormalities and diarrhea suggested the second mechanism, i.e., immune-mediated effects, as the most plausible underlying mechanism.

Our patient was also suspected to have macrolide-resistant *M. pneumoniae* (MRMP). First identified in Japan in 2001 [10], MRMP has since emerged as a global concern. While definitive diagnosis requires susceptibility testing or molecular analysis, these methods are often impractical in routine clinical practice. Therefore, MRMP is typically suspected when there is no clinical improvement after 72 hours of macrolide therapy [11]. In our patient, persistent fever and progressive pneumonia further supported this diagnosis. Corticosteroids have proven effective in managing refractory MPP, particularly when associated with elevated LDH levels [12,13]. The pronounced elevation of LDH in our patient likely reflects the severity of systemic inflammation and contributed to the development of intestinal involvement.

Additionally, high serum IgE levels, as seen in our patient with atopic dermatitis, may predispose individuals to extrapulmonary manifestations. Previous studies have reported a high prevalence of extrapulmonary complications of *M. pneumoniae* infections in patients with high serum IgE levels, suggesting that immune imprinting leading to IgE production could act as a trigger for the heterogeneous spectrum of clinical disorders associated with *M. pneumoniae* infection [14].

## Conclusions

This case highlights a rare presentation of *M. pneumoniae* infection, where abdominal pain was the predominant symptom. The patient’s condition required treatment with corticosteroids and tosufloxacin. The abdominal pain was attributed to intestinal involvement, a possibly extrapulmonary manifestation of *M. pneumoniae* infections. Clinicians must recognize that *M. pneumoniae* infection can occasionally present with abdominal pain as the predominant symptom, particularly in pediatric patients with minimal or no respiratory symptoms.
